# Overshadow Effect of Psl on Bacterial Response to Physiochemically Distinct Surfaces Through Motility-Based Characterization

**DOI:** 10.3389/fcimb.2018.00383

**Published:** 2018-10-29

**Authors:** Chunhui Zhai, Wenchao Zhang, Jingchao Zhang, Luyan Z. Ma, Kun Zhao

**Affiliations:** ^1^Key Laboratory of Systems Bioengineering (Ministry of Education), School of Chemical Engineering and Technology, Tianjin University, Tianjin, China; ^2^State Key Laboratory of Microbial Resources, Institute of Microbiology, Chinese Academy of Sciences, Beijing, China

**Keywords:** *P. aeruginosa*, biofilm, bacterial collective motility, Psl, surface motility

## Abstract

Biofilms of *Pseudomonas aeruginosa* are ubiquitously found on surfaces of many medical devices, which are the major cause of hospital-acquired infections. A large amount of work has been focused on bacterial attachment on surfaces. However, how bacterial cells evolve on surfaces after their attachment is the key to get better understanding and further control of biofilm formation. In this work, by employing both single-cell- and collective-motility of cells, we characterized the bacterial surface movement on physiochemically distinct surfaces. The measurement of cell surface motility showed consistent results that gold and especially platinum surfaces displayed a stronger capability in microcolony formation than polyvinyl chloride and polycarbonate surfaces. More interestingly, we found that overproduction of Psl led to a narrower variance in cell surface motility among tested surfaces, indicating an overshadow effect of Psl for bacteria by screening the influence of physicochemical properties of solid surfaces. Our results provide insights into how *Pseudomonas aeruginosa* cells adapt their motion to physiochemically distinct surfaces, and thus would be beneficial for developing new anti-biofouling techniques in biomedical engineering.

## Introduction

Biofilms are surface-associated multicellular communities in which microbial cells are embedded in extracellular polymeric matrices (Costerton et al., 1987; O'Toole et al., 2000; Hall-Stoodley et al., 2004; Oliveira et al., 2015). They are ubiquitously found on surfaces of various devices including medical implants (Manivasagam et al., 2010) and industrial equipment like tubes and engine cooling machines (Starosvetsky et al., 2007), which cause an increasing rate of hospital-acquired infections (Page et al., 2009; Cloutier et al., 2015) as well as biocorrosion of industrial equipment (Li et al., 2011). So bio-contamination of materials has received an extensive attention both in industries and in fundamental research.

*Pseudomonas aeruginosa* (*P. aeruginosa*) is one of the most common bacteria associated with device-related infections (Hall-Stoodley et al., 2004), and has been a model organism for biofilm studies. A generally accepted picture of biofilm formation follows a sequential development stages including initial attachment of bacterial cells on surfaces, microcolony formation and maturation, and finally dispersal of cells from matured biofilms to start a development cycle of next “generation”. Many bacterial factors that affect cell-surface interactions have been reported (O'Toole et al., 2000; Verran and Whitehead, 2005; O'Toole and Wong, 2016). For example, G. O'Toole and R. Kolter showed that the flagellar mutant of *P. aeruginosa* was defective for surface attachment (O'Toole and Kolter, 1998). D. Woods et al. found that type IV pili (TFP) were important for the adherence of *P. aeruginosa* to eukaryotic cell surfaces (Woods et al., 1980). Besides motility appendages, Psl, which is one type of exopolysaccharide consisting of a repeating penta-saccharide including D-mannose, D-glucose, and L-rhamnose (Ma et al., 2007), has been shown to greatly promote *P. aeruginosa* surface adhesion (Ma et al., 2006; Mann and Wozniak, 2012). After attachment, *P. aeruginosa* cells could move on the surface in a TFP-driven twitching mode and form microcolonies, which can then grow into a matured biofilm with mushroom-like structures (Davies et al., 1998; Stoodley et al., 2002; Klausen et al., 2003). Two different twitching modes have been reported. One is crawling in which cells lie down on a surface and move parallel to the surface; the other is walking in which cells stand up and move on a surface in a vertical fashion (Gibiansky et al., 2010; Conrad et al., 2011). In addition, *P. aeruginosa* could also do a slingshot motion on soft surfaces using TFP (Jin et al., 2011; Zhang et al., 2014). It has been suggested that *P. aeruginosa* could employ different surface motility strategies in varying nutrition conditions (Ni et al., 2016). Interestingly, bacterial surface exploration pattern could also be affected by Psl through a Psl-guided rich-get-richer mechanism (Zhao et al., 2013), implying the crucial role of Psl for biofilm development, particularly at early stages including attachment to solid surfaces and microcolony formation.

From the aspect of solid surfaces, the physicochemical properties of surfaces such as roughness and surface charges have also been shown to affect microbial adhesion (Dexter et al., 1975; Díaz et al., 2007; Rzhepishevska et al., 2013; Bohinc et al., 2016), and methods based on controlling the surface properties through surface modifications both physically and chemically have already been proposed (Cloutier et al., 2015) to prevent bacterial surface attachment. For instance, surfaces could be modified by antimicrobial peptides and biofilm-dispersing enzymes to prevent biofouling by killing attached bacteria (Alves and Olívia Pereira, 2014; Gallarato et al., 2017). The topological structures of surfaces such as certain nano-structures or micro-structures were also demonstrated to have a positive effect on hindering bacterial cell-cell and cell-surface interactions (Díaz et al., 2007; Bohinc et al., 2016). Although a large amount of work has been done, our understanding on how bacteria respond to surfaces with different physiochemical properties, particularly on how bacterial surface motility changes at a single-cell level, is still very limited.

In this work, using a state-of-art high-throughput bacterial tracking technique, we developed a method to fully characterize the bacterial surface motility at a single-cell resolution by combining multiple quantitative parameters for both single-cell dynamics and collective motion of cells. Using the developed method, we measured the surface motility of *P. aeruginosa* on five chosen surfaces: glass, polycarbonate (PC, also known as Makrolon), polyvinyl chloride (PVC), gold, and platinum, which are commonly used in medical devices and daily supplies. The results showed bacteria behaved differently in their surface motility in response to physiochemically distinct surfaces. More interestingly, we found that overproduction of Psl led to a narrower variance in cell surface motility among tested surfaces, indicating an overshadow effect of Psl for bacteria by screening the influence of physicochemical properties of solid surfaces. Our results provide insights for designing new anti-biofouling surfaces.

## Materials and methods

### Surface preparations

Cover glasses (Leibusi), PC (Dongguan Lingmei New Material), and PVC (Dongguan Lingmei New Material) slices were washed first by ethanol and then by deionized water each for three times. Gold and platinum surfaces were prepared by sputter-coating a ~10 nm gold and platinum layer on cover glasses, respectively. The characterized surface properties were summarized in Supplementary Table S1. Water contact angle (WCA) and diiodomethane contact angle (DCA) measurements (Supplementary Figures S1, S2) were performed on a contact angle meter (JC2000D, Powereach). The sessile drop technique was employed to measure contact angles of water and diiodomethane on five surfaces and to calculate the solid surface energies based upon the Owens/Wendt theory (Owens and Wendt, 1969). Surface zeta potential measurements were performed on a surface zeta potential tester (SurPASS, Anton Paar) with a 5 mM KCl solution at pH = 5.5. Atomic force microscopy (AFM5500, Agilent Technologies Inc.,) was used for the characterization of the surface morphology of materials (Supplementary Figure S3).

### Bacterial strains, culture conditions and flow cell experiments

Wild-type *P. aeruginosa* strain PAO1 and its isogenic mutants ΔP_*psl*_/P_*BAD*_-*psl* and Δ*pslBCD* were used in this study (Ma et al., 2006). For ΔP_*psl*_/P_*BAD*_-*psl*, 1% (w/v) arabinose was used under which Psl would be overproduced. Wild-type *P. aeruginosa* c-di-GMP reporter strain (courtesy of Prof. Fan Jin at University of Science and Technology of China), containing transcriptional pcdrA::gfp fusion plasmids was used to estimate the Psl production activity by measuring the GFP fluorescence of those cells. Strains were grown on LB agar plates at 37°C for 20 h. Monoclonal colonies were inoculated into culturing tubes containing 5 mL of FAB medium [2 g L^−1^ (NH_4_)_2_SO_4_, 9 g L^−1^Na_2_HPO_4_·7H_2_O, 3 g L^−1^KH_2_PO_4_, 3 g L^−1^ NaCl, 93 mg L^−1^ MgCl_2_, 14 mg L^−1^ CaCl_2_·2H_2_O, 1 ml L^−1^ Trace metals solution (200 mg L^−1^ CaSO_4_·2H_2_O, 200 mg L^−1^ FeSO_4_·7H_2_O, 20 mg L^−1^ MnSO_4_·H_2_O, 20 mg L^−1^ CuSO_4_·5H_2_O, 20 mg L^−1^ ZnSO_4_·7H_2_O, 10 mg L^−1^ CoSO_4_·7H_2_O, 10 mg L^−1^ Na_2_MoO_4_·2H_2_O, 5 mg L^−1^ H_3_BO_3_)] with 30 mM glutamate and then incubated on a shaker at 37°C. Bacterial cultures were harvested at the exponential growth phase (OD_600_ ≈ 0.5). A diluted culture (OD_600_ ≈ 0.01) was used for flow cell experiments.

Flow cells were purchased from Denmark Technical University, and were assembled as previously described (Sternberg and Tolker-Nielsen, 2006). FAB medium containing 0.6 mM glutamate was used for flow cell experiments. After bacteria inoculation, a 15-min incubation period was allowed to let bacterial cells attach to the bottom surface, which then was followed by initiating a continuous flow at 3 mL/h and image recording for 12~24 h at 30°C.

### Data collection and image analysis

Images were captured using an EMCCD camera (Andor iXon Ultra 888) on a Leica DMi8 microscope equipped with a Zero Drift autofocus system. The image size is 66.5 μm × 66.5 μm (1,024 × 1,024 pixels). Bright-field images were recorded every 3 s. For fluorescent images, GFP filter (460–500 nm exciter and 512–542 nm emitter) was used for detection of GFP fluorescence. Fluorescent images were taken right after bacteria were inoculated into the flow cell and more than 1,000 cells were imaged. For each condition, at least three repeats were performed, and for bright-field recordings each repeat generated more than 14,400 images.

Bright-field images were analyzed using the same method as previous described (Zhao et al., 2013). Simply speaking, 16-bit grayscale images were first converted to binary images for the detection of bacteria with a standard image processing algorithm. Geometry information of cells such as center position, size and aspect ratio etc. were then collected. Bacterial trajectories were obtained by connecting cell positions in all frames of a time series, from which bacterial motion can be measured and analyzed. For characterization of single-cell motility on surfaces, to minimize the effect of cell-cell collision on single-cell movement, we only used cell motility data at early stages of biofilm formation, when the number of cells was still small (less than 60 cells in the field of view) and cells were sparsely distributed on surfaces. The fluorescence reporter intensity of each cell in the fluorescent images was determined by the total fluorescence inside the area of the cell divided by the area of the cell.

For instantaneous speed measurement, the trajectories of cells were smoothed with a 21-point Savitzky-Golay filter. The crawling or walking/standing status of a bacterial cell was classified by the aspect ratio of the projected cell image (length/width). Cells are in crawling status when they have an aspect ratio ≥2, and in walking/standing status when they have an aspect ratio < 2. The mean square displacement (MSD) of cells was calculated as $\langle \Delta r^{2}(\Delta t)\rangle =\langle {\left[r\left(t_{0}+\Delta t\right)-\;r(t_{0})\right]}^{2}\rangle$, here ***r***(*t*_0_) is the position vector of a cell at time *t*_0_, and Δ*t* represents the time interval. Surface coverage and bacterial visit frequency analysis were done in the same way as in previous work (Zhao et al., 2013). Simply speaking, the surface coverage was generated from the bacterial trajectories using the area of each tracked cell. The visit frequency distributions were generated at full data resolution; for the visit maps, the center of the cell was used to mark the trail and each of these marks were spread over a square patch with eight pixels (~0.5 μm) wide, in accordance with bacterium width.

A cluster is an aggregation of multiple cells. We used a minimum distance criterion to judge whether a cell belongs to a cluster or not. If the minimum distance between any point of the scrutinized cell body and any point of any cell body of the cluster, is smaller than 0.5 μm (i.e., about one width of a bacterial cell), then the scrutinized cell is considered to belong to the cluster.

## Results

### Characterization of single-cell motility of cells on the five chosen surfaces

**Figure 1:**
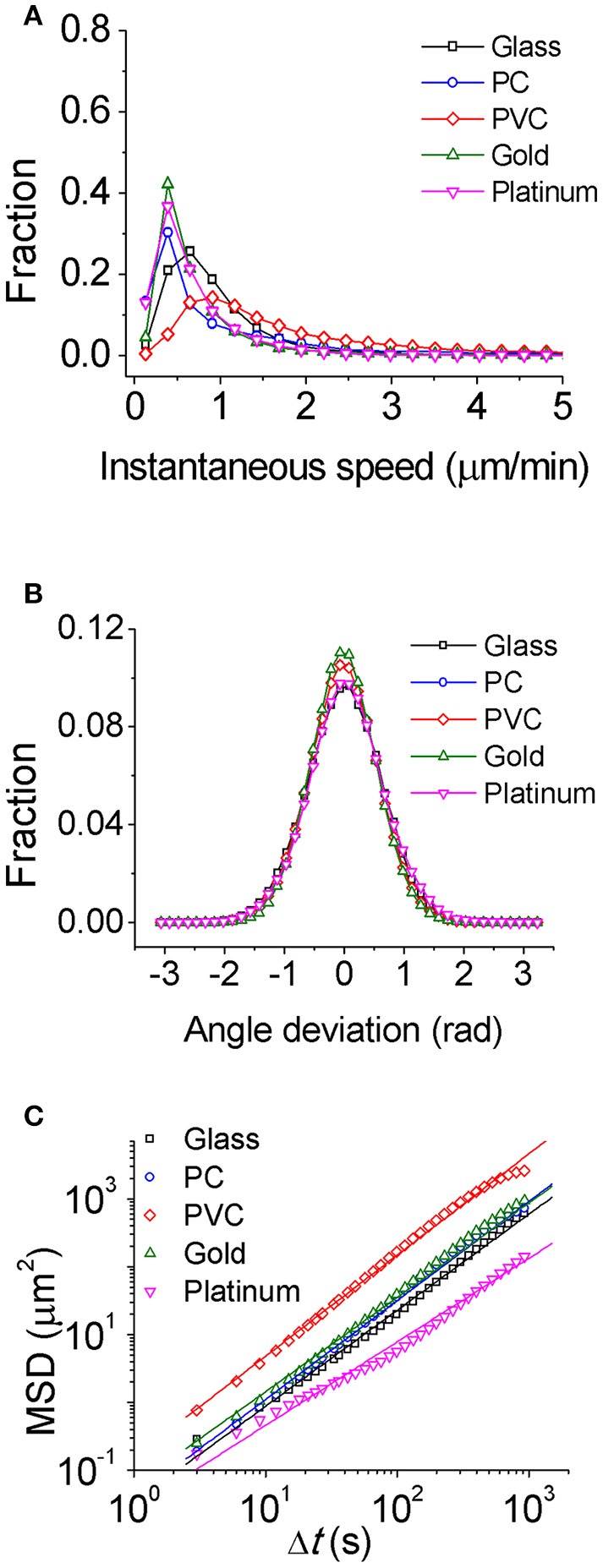
Characterization of single-cell motility for PAO1 WT on the five chosen surfaces. **(A)** Instantaneous speed distributions. **(B)** Distributions of angle deviation. **(C)** Mean square displacements (MSD) of cells. The slope of MSD is 1.40 for glass, 1.42 for PC, 1.46 for PVC, 1.38 for gold, and 1.23 for platinum, respectively. The number of cells used for statistical analysis is 407 for glass, 384 for PC, 369 for PVC, 177 for gold, and 197 for platinum, respectively.

To study how bacteria respond to physiochemically distinct surfaces, we first characterized single-cell motility of cells. Figure 1A displays the distribution of instantaneous speed of cells on different surfaces. It shows that among the five tested surfaces, the PVC surface has a relatively broad distribution of cell instantaneous speed with the highest peak value of 0.88 μm/min, and the glass surface has a less broad distribution with a peak value of 0.64 μm/min, while the PC, gold, and platinum surfaces all have a similar narrow distribution with a peak value of 0.39 μm/min. The average of instantaneous speed from high to low follows the order of PVC (1.8 μm/min) > PC (0.97 μm/min) > glass (0.86 μm/min) > gold (0.72 μm/min) > platinum (0.65 μm/min). Thus, among the tested surfaces, cells on the PVC surface moved fastest, whereas cells on the platinum surface moved slowest with a speed of about 1/3 of that on the PVC surface. The deviation angle of cell motion, which is defined as the angle between its cell body axis and the moving direction, was also measured. And the results show not much difference among tested surfaces (Figure 1B).

Alternatively, MSD of cells could be also used to characterize single-cell motion. MSD measures to what extent a cell motion deviates from a typical random diffusive motion. The slope of MSD curve in a log-log scale reflects the shape of the corresponding trajectory with value of 1 indicating a random diffusive behavior and value of 2 indicating geometrically straight motion. The slopes of MSD curves from high to low are PVC (1.5) > PC (1.4) ~ glass (1.4) ~ gold (1.4) > platinum (1.2) (Figure 1C). They are all above 1 indicating that the cell motion on all tested surfaces is on average super-diffusive. This trend is consistent with the average speed measurement.

### Characterization of collective motion of cells on the five chosen surfaces

**Figure 2:**
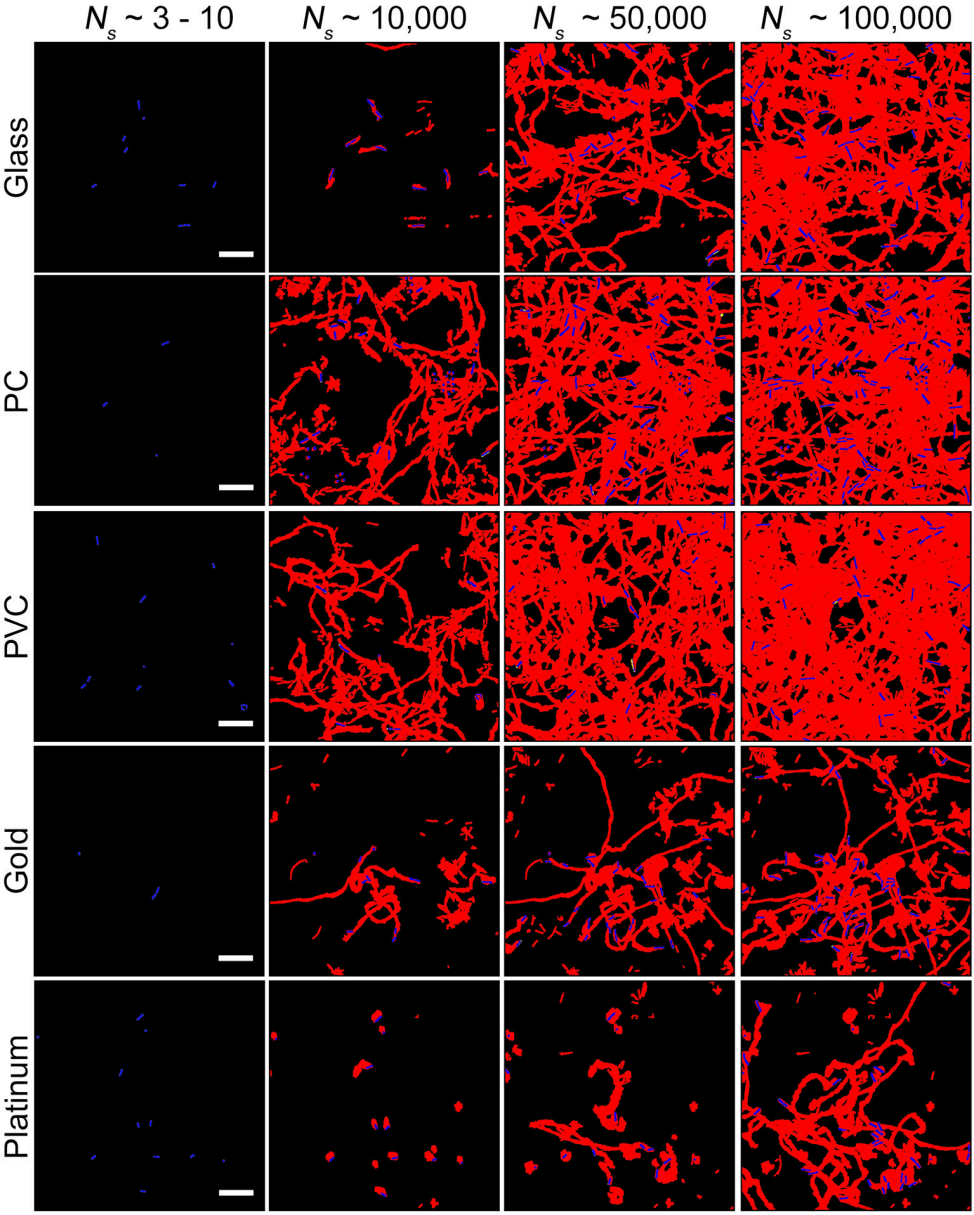
Surface coverages at different total number of bacterial visits *N*_*s*_ for the five tested surfaces. Red and black colors represent contaminated (i.e., visited by bacteria) and fresh areas, respectively. Bacteria in the current frame are shown in blue. Scale bars are 10 μm.

Considering that biofilm development is a process involving collective motion of multiple cells, here we employed a method based on collective motion to study how cells change their surface exploration patterns in response to surfaces with distinct physicochemical properties. First, we measured the efficiency of surface coverage. Figure 2 shows an evolution of surface coverage of bacterial visits at different time points. Red color represents the areas that have been visited or contaminated by bacteria, whereas black color indicates that the areas are fresh and have not been visited by bacteria. Surface coverages of different tested surfaces are compared at a same total number of bacterial visits *N*_*s*_(*N*_s_ ≡ ∑ *n*_*i*_, where *n*_*i*_ is the number of bacteria in frame *i*) to minimize the effect of initial inoculation conditions (Zhao et al., 2013). The results show that surface coverages of the PVC and PC surfaces increase much faster than those of the gold and platinum surfaces. At *N*_*s*_~ 100,000, the PVC and PC surfaces have surface coverages of 50~65% that have been visited by bacteria. In contrast, the gold and platinum surfaces have much less surface coverages of 25~40% at the same *N*_*s*_. So, in terms of surface coverage, cell motion is more efficient on the PVC and PC surfaces than on the gold and platinum surfaces.

It has been shown that bacteria could use a Psl-based rich-get-richer mechanism to coordinate their surface motion (Zhao et al., 2013), in which surface spots with a high concentration of Psl attracted bacteria to visit more frequently, and more bacterial visits in turn enhanced the Psl concentration of those spots further, thus a positive feedback loop was formed and led to microcolony formation. The resulted distribution of bacterial visits could be approximately expressed by a power law. In this work, to quantitatively characterize the bacterial collective motility, we used the exponent of power law as a metric.

**Figure 3:**
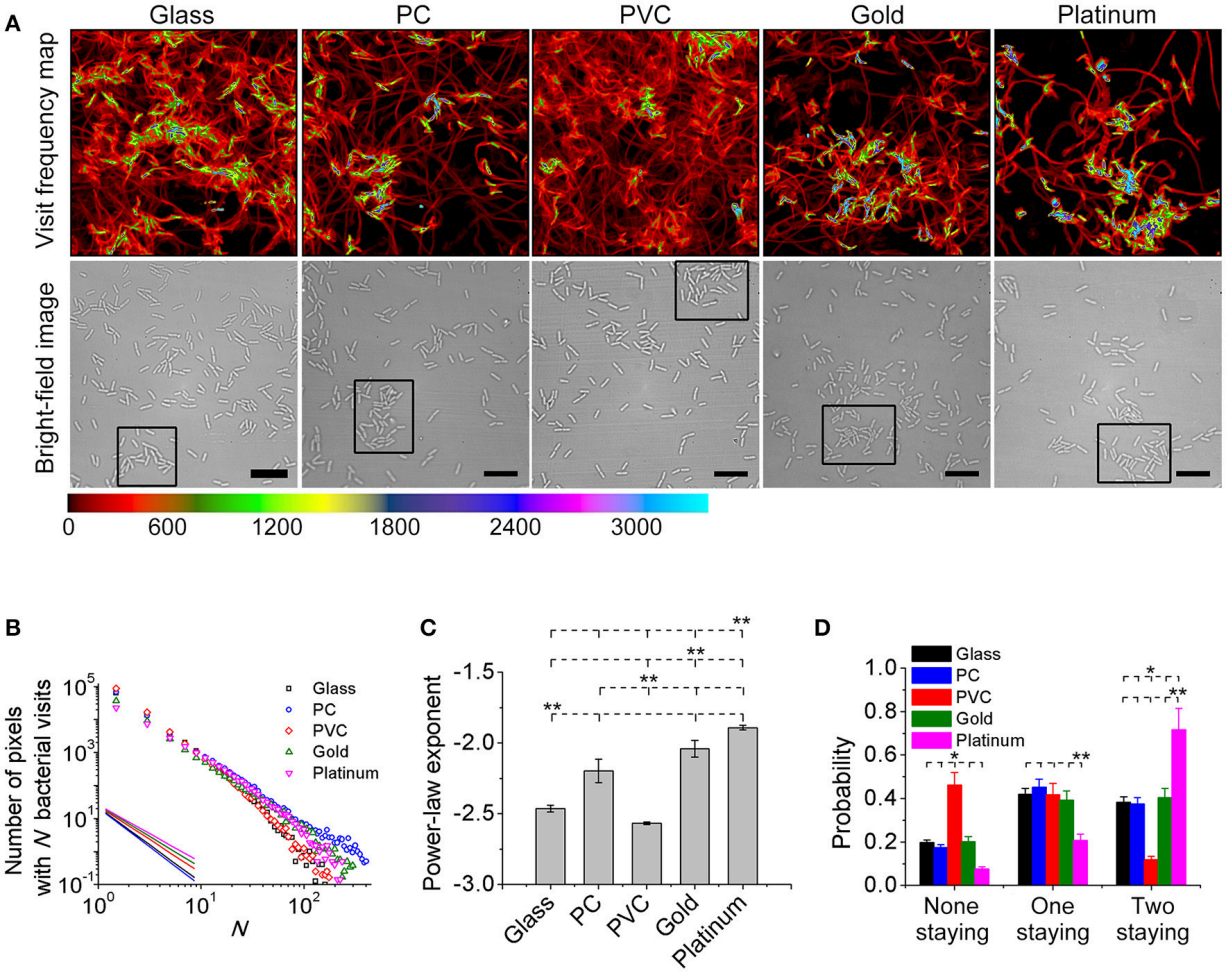
Visit frequency distributions and post-division cell fates on different surfaces. **(A)** Visit frequency maps (top row) and bright-field images (bottom row) at total bacterial visits of 424,000 for glass, 393,800 for PC, 439,100 for PVC, 378,900 for gold, and 291,000 for platinum, respectively, when microcolonies are just starting to form (examples of first microcolonies in the field of view defined as clusters of ~ 25 cells are outlined by black rectangles in the bright-field images). **(B)** Visit frequency distributions. Solid lines show power-law decay. **(C)** Power-law exponents obtained from **B**. Error bars are standard deviations of three repeats. **(D)** Probabilities of post-division cell fates. Error bars are estimated from 1/√*N*_div_, where *N*_div_ is the total number of division events during the period of interest (*N*_div_ ≥ 100). Statistical significances are measured using unbalanced one-way ANOVA (Kruskal-Wallis test for distribution medians) and multiple comparison tests using Tukey's honest significant difference criterion with a 0.05 significant value. *p < 0.1 and **p < 0.05. The position of each asterisk indicates the reference material used in the statistical analysis. Scale bars are 10 μm.

Toward this end, a bacterial visit frequency map was first constructed from all bacterial trajectories. Figure 3A shows the bacteria visit frequency maps for cells on the glass, PC, PVC, gold, and platinum surfaces for a period from inoculation of cells to the time when microcolonies began to form (in this work, we defined microcolonies as clusters of more than 25 cells, see Materials and Methods for cluster definition). Supplementary Figure S4 illustrates more developed microcolonies 1 h later at the same locations as in Figure 3A, which confirm the growth of microcolonies. Then the distribution curves of bacterial visits were obtained through a histogram analysis and were fitted with a power law (Figure 3B). The exponents of the power law are measured to be −2.6 ± 0.01 (PVC) < −2.5 ± 0.03 (glass) < −2.1 ± 0.08 (PC) < −2.0 ± 0.06 (gold) < −1.9 ± 0.02 (platinum) (mean ± standard deviation of three repeats) (Figure 3C). Using unbalanced one-way ANOVA (Kruskal-Wallis test for distribution medians) and multiple comparison tests using Tukey's honest significant difference criterion with a 0.05 significant value, the differences in the measured exponents for different tested surfaces are statistically significant. Among the five tested surfaces, the visit distribution on the platinum surface follows a power law with the least-negative exponent, indicating a more hierarchical distribution, whereas the one on the PVC surface shows a power law with the most-negative exponent, implying a more uniform distribution. By counting the total number of visits at which the first microcolony appeared in the field of view, which are 439,100 for PVC, 424,000 for glass, 393,800 for PC, 378,900 for gold, and 291,000 for platinum, we can see that more hierarchical distribution of bacterial visits corresponds to less total number of visits required for microcolony formation. This is also consistent with observations in reference (Zhao et al., 2013).

To gain better understanding of the observed different behavior in microcolony formation on different surfaces, we investigated post-division cell fates. A post-division cell is defined as “staying” if the cell's next division event is still detected in the field of view, and “leaving” if otherwise. For each cell division event, there are three possible fates of two post-division cells: both leaving, both staying or one staying and the other leaving. Figure 3D shows the measured probabilities of three fates by collecting all observed division events. For both cells staying, the probability is highest (~72%) on the platinum surface, and is lowest (~12%) on the PVC surface. The results for both leaving are just opposite, where the probability is lowest (~12%) on the platinum surface but highest (~46%) on the PVC surface. For the case of one staying and the other leaving, the probabilities on the glass, PC, PVC, and gold surfaces are comparable although it is slightly higher on the PC surface. The probability on the platinum is lowest (~21%). So cell division contributes more to the increase of cell population on the platinum surface than that on the PVC surface. Interestingly, this implies that cells may deploy different strategies for colonization on different surfaces. For cells on the platinum surface, most daughter cells would stay after each cell division, and the daughter cells continue to grow and divide, which will then result in an exponential growth of cell population in a region centered around founder cells (i.e., region of microcolonies). This fast exponential growth is advantageous for cells to occupy the resources of nearby environment to survive. On the contrary, for cells on the PVC surface, since post-division cells have a low tendency to both stay, the increase of cell population and hence microcolony formation is slow, so they don't have advantages in the competition for resources with those cells that can quickly form microcolonies. However, the efficiency of surface coverage is high on the PVC surface, and then with a large part of post-division cells either detaching or moving out to other places, they might have better chances to colonize new surfaces.

### Effect of psl on the variance of bacterial surface exploration among the tested surfaces

Psl has been shown to be important in both promoting cell-surface adhesion and regulating cell surface motility (Ma et al., 2006; Zhao et al., 2013; O'Toole and Wong, 2016). So we hypothesize that Psl would affect the response of cells to physiochemically distinct surfaces, and if the Psl-mediated interactions could get enhanced, the influence of physicochemical properties of different solid surfaces might be overshadowed.

To test this hypothesis, we used two Psl mutant strains. One is Psl- strain Δ*pslBCD*, which cannot produce Psl, and the other is Psl-overproducing (Psl++) strain ΔP_*psl*_/P_*BAD*_-*psl*, whose Psl production is controlled using arabinose. In this study, 1% arabinose was added for ΔP_*psl*_/P_*BAD*_-*psl* and Psl was overproduced under this condition.

**Figure 4:**
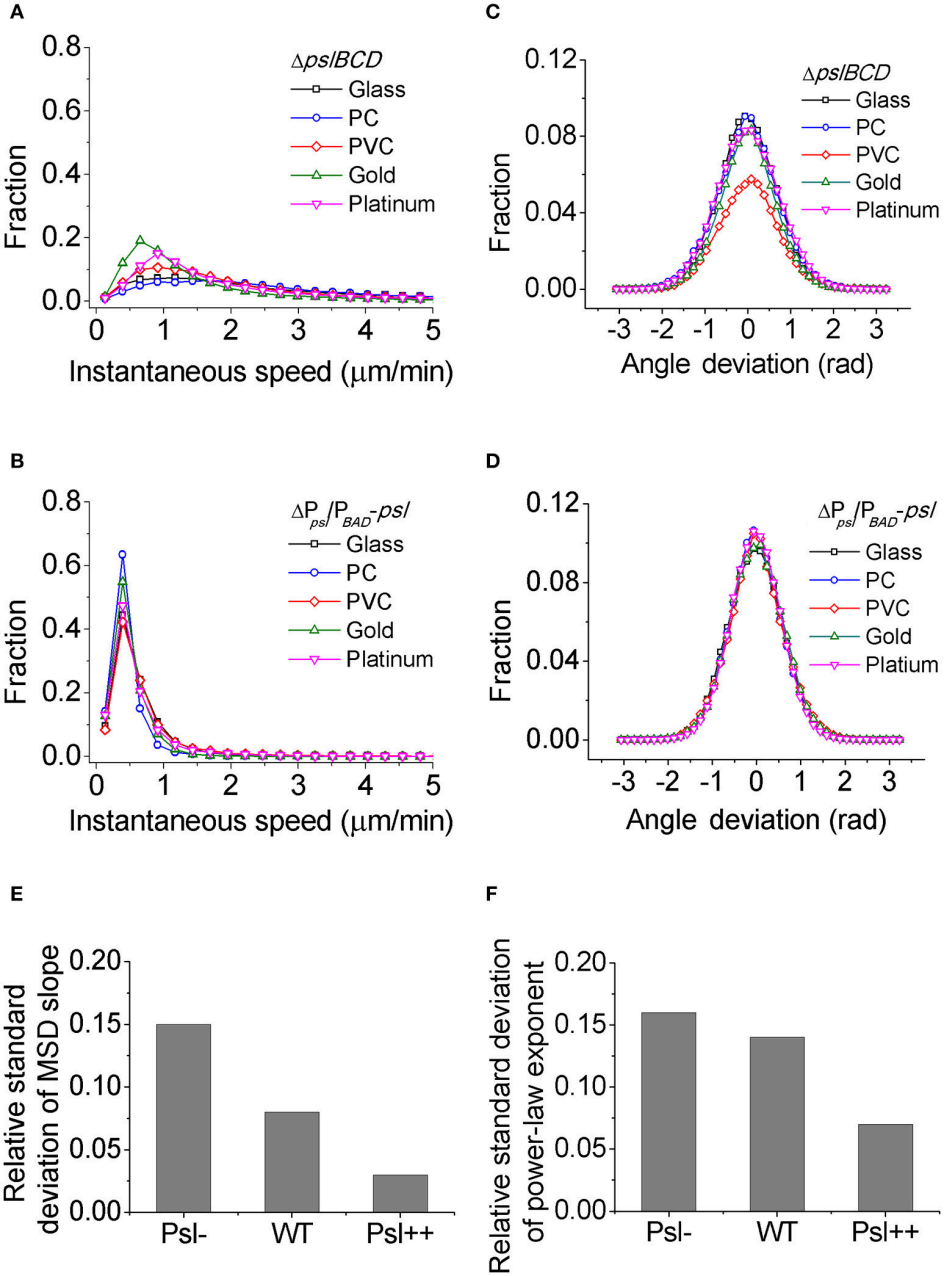
The distributions of speed and angle deviation, the relative standard deviations of MSD slope and power-law exponent for different strains. **(A)** and **(B)** are speed distributions for Psl- (Δ*pslBCD*) and Psl++ (ΔP_*psl*_/P_*BAD*_-*psl* with 1% (w/v) arabinose addition in the medium), respectively. **(C)** and **(D)** are angle deviation distributions for Psl- and Psl++, respectively. **(E)** and **(F)** are the relative standard deviations of MSD slope and power-law exponent, respectively, for Psl-, WT and Psl++.

For each mutant strain, the single-cell- and collective- motility of cells on different surfaces were characterized in the same way as for WT cells (Supplementary Figures S5–S8). In order to see the effect of Psl on the variance of bacterial surface exploration, we compared the motility-characterization results of cells for three strains. Figure 4 shows the distributions of both speed and angle deviation of cells on the tested surfaces (Figures 4A–D). We can see that the variance in the speed distribution of cells among the tested surfaces is relatively larger for Psl- cells than for Psl++ cells. The distribution of angle deviation is also similar. To quantitatively characterize the variation of single-cell motility, we first measured the MSD slopes for each mutant on the five tested surfaces (summarized in Table 1), and then calculated the relative standard deviation of the obtained MSD slopes (defined by the standard deviation divided by the mean of MSD slopes). Figure 4E shows the result. It demonstrates that the variation among the tested surfaces for three strains follows the order of (from large to small) Psl- > WT > Psl++. Similarly, a relative standard deviation of power-law exponent among the five surfaces were calculated to quantitatively characterize the variation of collective-motility (Figure 4F, Table 2). The result shows the same trend as for the MSD slope: the variation of power-law exponent decreases with the increase of cells' Psl production. All these results clearly support our hypothesis that Psl can affect the response of cells to distinct surfaces, and overproduction of Psl could overshadow the influence of physicochemical properties of different surfaces. This would lend cells a tool to adapt to physiochemically distinct surfaces by adjusting their Psl production to reduce the direct effect from physicochemical properties of solid surfaces. Thus, this overshadow effect would be worth taking into consideration when designing anti-fouling surfaces.

**Table 1:** MSD slopes of Psl-, WT, and Psl++ on the five chosen surfaces.

	**Glass**	**PC**	**PVC**	**Gold**	**Platinum**
Psl-	1.2 ± 0.01	1.1 ± 0.002	1.4 ± 0.05	1.0 ± 0.15	1.0 ± 0.08
WT	1.4 ± 0.02	1.4 ± 0.23	1.5 ± 0.04	1.4 ± 0.02	1.2 ± 0.15
Psl++	1.4 ± 0.02	1.4 ± 0.003	1.5 ± 0.09	1.4 ± 0.11	1.4 ± 0.20

**Table 2:** Power-law exponents of Psl-, WT, and Psl++ on the five chosen surfaces.

	**Glass**	**PC**	**PVC**	**Gold**	**Platinum**
Psl-	−3.6 ± 0.02	−3.7 ± 0.06	−2.6 ± 0.01	−2.8 ± 0.02	−3. 2 ± 0.02
WT	−2.5 ± 0.03	−2.1 ± 0.08	−2.6 ± 0.01	−2.0 ± 0.06	−1.9 ± 0.02
Psl++	−1.9 ± 0.05	−1.7 ± 0.21	−1.9 ± 0.14	−1.6 ± 0.10	−1.7 ± 0.12

## Discussion

Our findings showed that *P. aeruginosa* PAO1 behaved differently in their surface motility in response to varying surface conditions. The measurements of both single-cell- and collective-motility showed consistent results which implied a higher tendency to form microcolonies (hence biofilms) on the gold and especially platinum surfaces than on the plastic and glass surfaces. This is consistent with an earlier work on *Helicobacter pylori*, which showed that large agglomerates of *Helicobacter pylori* cells tended to form more on copper surfaces compared with other tested polyvinyl chloride, polypropylene and glass surfaces (Azevedo et al., 2006).

A deep and more fundamental question is what the mechanisms are for cells to sense and respond to different solid surfaces. *Vibrio cholera* has at least three types of pilus that can interact with chemically distinct surfaces (Reguera and Kolter, 2005). How *P. aeruginosa* sense physiochemically distinct surfaces? The current understanding of *P. aeruginosa* surface sensing involves both chemical cues such as nutrient availabilities, and exopolysaccharides as well as physical cues such as flagellar- or TFP-mediated sensing (O'Toole and Wong, 2016). In this work, we investigated the role of Psl in cell response to different solid surfaces. Our results showed that the relative standard deviation of surface motility decreased with the Psl production of cells, indicating that overproduction of Psl can overshadow the influence of distinct physicochemical properties of surfaces. These results provide additional cues to the above fundamental question in two aspects. On one hand, since bacteria can regulate Psl production via surface sensing mechanism such as Wsp in *P. aeruginosa*, a chemotaxis-like system that can regulate c-di-GMP levels (Güvener and Harwood, 2007; Hickman and Harwood, 2008; O'Connor et al., 2012), cells might be able to increase the Psl production to screen the influence of distinct physicochemical properties of surfaces, similar to the behavior of Psl++ cells that we tested. However, to test this hypothesis, more work using mutants on the Wsp system will be needed. On the other hand, if the Psl production of *P. aeruginosa* WT cells is heterogeneous (*i.e*., the Psl production varies from cell to cell), bacterial cells that would finally colonize a surface could be passively selected by the physicochemical properties of the surface based on cells' Psl production activities. From a natural selection point of view, by doing this way, bacterial community would benefit from the heterogeneity in the Psl production of cells.

It has been shown that isogenic populations of bacterial cells display significant phenotypic variation such as in growth, stress response, and cell morphologies even under same environmental conditions, which result partially from both stochasticity in gene expression and also fluctuations in other cellular components (Elowitz et al., 2002; Ackermann, 2015; Evans and Ling, 2018). For example, T. Vissers et al. showed a strong phenotypic heterogeneity in the surface attachment of *E. coli* cells (Vissers et al., 2018). They found that among all analyzed cells, some cells remained non-attached while others could adhere to the surface but with varied adhesion strength.

**Figure 5:**
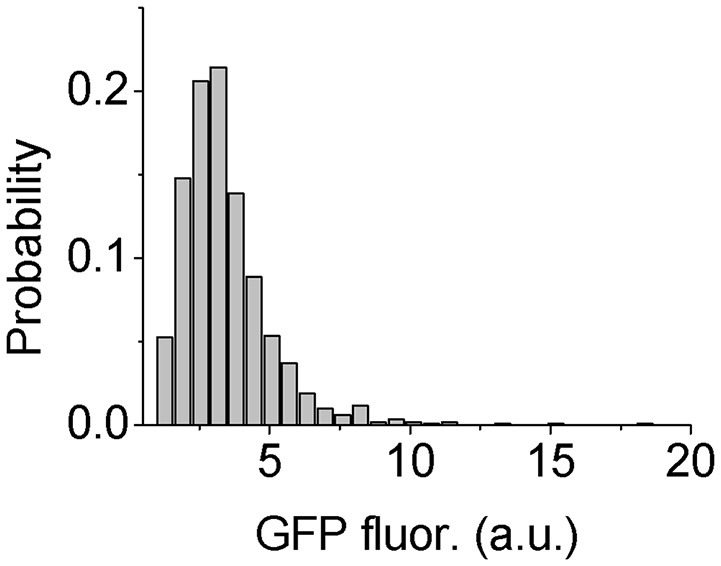
Distribution of GFP fluorescence of reporter cells. The number of cells used for statistical analysis is 1101.

However, to test the heterogeneity of Psl production in *P. aeruginosa* cells, we need to find a way to quantitatively measure the Psl production activity. It has been shown that the high level of c-di-GMP stimulates the production of exopolysaccharide Psl as well as the extracellular matrix adhesion protein CdrA in *P. aeruginosa* (Hickman et al., 2005; Borlee et al., 2010; Irie et al., 2012). Based on this correlation, Y. Irie et al. constructed c-di-GMP reporter strains containing transcriptional p*cdrA*::*gfp* fusion plasmids, in which the fluorescence of reporter cells was shown to be positively correlated with their Psl production (Irie et al., 2012). So here we used *P. aeruginosa* WT reporter strain to estimate the Psl production activity by measuring the GFP fluorescence of those cells. The results show clearly a broad distribution of fluorescence reporter intensity among cells, indicating heterogeneous production activity of Psl (Figure 5). This result is consistent with the recent published work by S. Yang et al. where they showed the heterogeneous production of Psl in planktonic *P. aeruginosa* cells using a different inverted reporter mutant in which the Psl expression level could be monitored via negatively correlated EGFP fluorescence (Yang et al., 2018). Given such variation in the Psl production activity, it is conceivable that when WT cells contact with surfaces, some cells with higher Psl production would be less affected by the physicochemical properties of solid surfaces. Thus even if the surface is otherwise not suitable for adhesion based on bare cell body condition (i.e., no Psl on bacterial cell envelop), those cells with higher Psl production would have a relatively large probability to attach to and then colonize it. This would contribute to the failure of many anti-biofouling surfaces.

Compared with cells having higher Psl production, those cells with less Psl production would be more sensitive to physiochemically distinct surfaces and are thus vulnerable to environmental changes. This also agrees with the “bet-hedging” mechanism (Philippi and Seger, 1989) proposed in early literature on phenotypic heterogeneities (Ackermann, 2015).

## Conclusion

In this study we have provided a comprehensive characterization of bacterial surface motility using both single-cell- and collective-motility on different surfaces. Compared with the PC and PVC surfaces, bacteria on the gold and platinum surfaces moved slower and showed a lower efficiency of surface coverage and a less-negative power-law exponent, which then resulted in a higher capability in microcolony formation. The results implied that the gold and platinum surfaces may have a higher tendency to be contaminated by bacteria than the PC and PVC surfaces.

We have also showed that Psl played an important role in bacterial response to different surfaces, and overproduction of Psl could overshadow the influence of physicochemical properties of solid surfaces. But more work is needed to reveal the regulating mechanism underlying it. In addition, for the future work, it will be also very helpful to test other culture conditions such as different medium and/or even other species to see whether the phenomena observed in this work persist and whether the exopolysaccharides secreted by other species could have similar effect on their surface colonization.

This study demonstrated a method to fully characterize bacterial surface motility. The findings provide insights into how *P. aeruginosa* adapt their motion to surfaces with different physicochemical properties, and thus would be beneficial for developing new anti-biofouling techniques in biomedical engineering.

## Author contributions

KZ conceived the project. CZ and KZ designed studies. CZ and JZ performed the experiments. LM constructed strains. CZ, WZ, and KZ analyzed the data. CZ, WZ, and KZ interpreted the data. CZ and KZ wrote the manuscript. All authors commented on the manuscript.

### Conflict of interest statement

The authors declare that the research was conducted in the absence of any commercial or financial relationships that could be construed as a potential conflict of interest.
