# Supplementary material for: Overshadow Effect of Psl on Bacterial Response to Physiochemically Distinct Surfaces Through Motility-Based Characterization
Source: Front Cell Infect Microbiol. 2018 Oct 29;8:383. doi: 10.3389/fcimb.2018.00383 (PMC6215810; doi:10.3389/fcimb.2018.00383)
Supplement: Supplementary file 1 [file Data_Sheet_1.PDF]

Supplementary Material

Overshadow effect of Psl on bacterial response to physiochemically distinct surfaces through motility-based characterization

Chunhui Zhai[1], Wenchao Zhang[1], Jingchao Zhang[1], Luyan Ma[2], Kun Zhao[1,*]

[1]Key Laboratory of Systems Bioengineering (Ministry of Education), School of Chemical Engineering and Technology, Tianjin University, Tianjin 300354, China

[2]State Key Laboratory of Microbial Resources, Institute of Microbiology, Chinese Academy of Sciences, Beijing 100101, China

* Correspondence: K Zhao (kunzhao@tju.edu.cn)

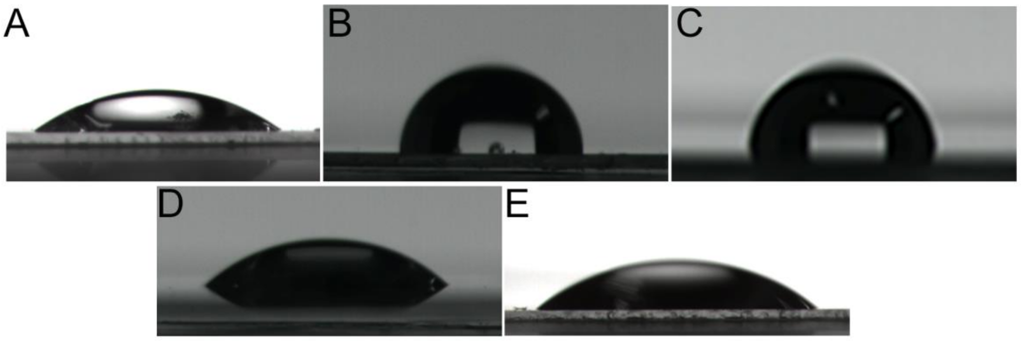

Supplementary FIGURE S1. The contact angles of water on the glass (**A**), **PC** (**B**), **PVC**(**C**), **gold** (**D**) **and platinum** (**E**) **surfaces.** The measured results are 15.7° on the glass, 86.4° on the PC, 76.6° on the PVC, 34.7° on the gold and 36.1° on the platinum surfaces.

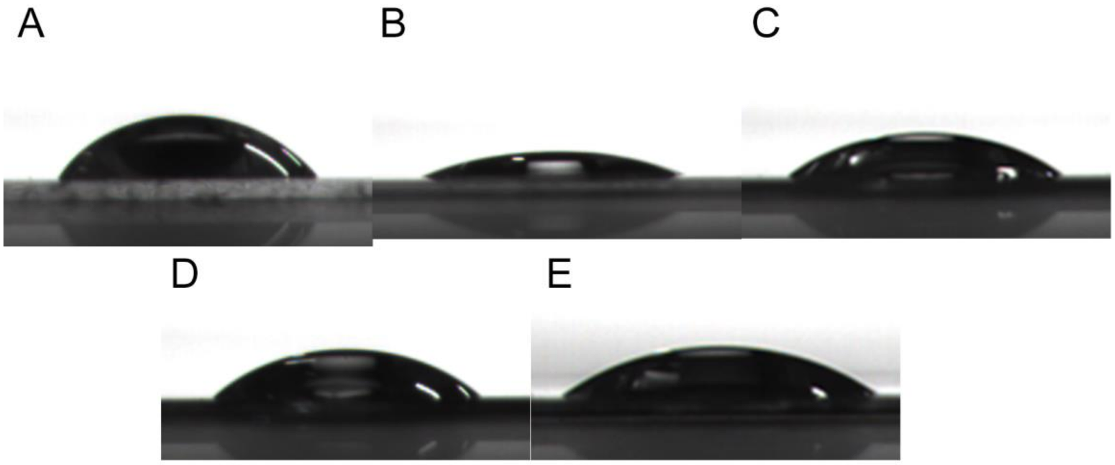

Supplementary FIGURE S2. The contact angles of diiodomethane on the glass (A), PC (B), PVC(C), gold (D) and platinum (E) surfaces. The measured results are 52.9° on the glass, 22.8° on the PC, 38.6° on the PVC, 48.6° on the gold and 41.8° on the platinum surfaces.

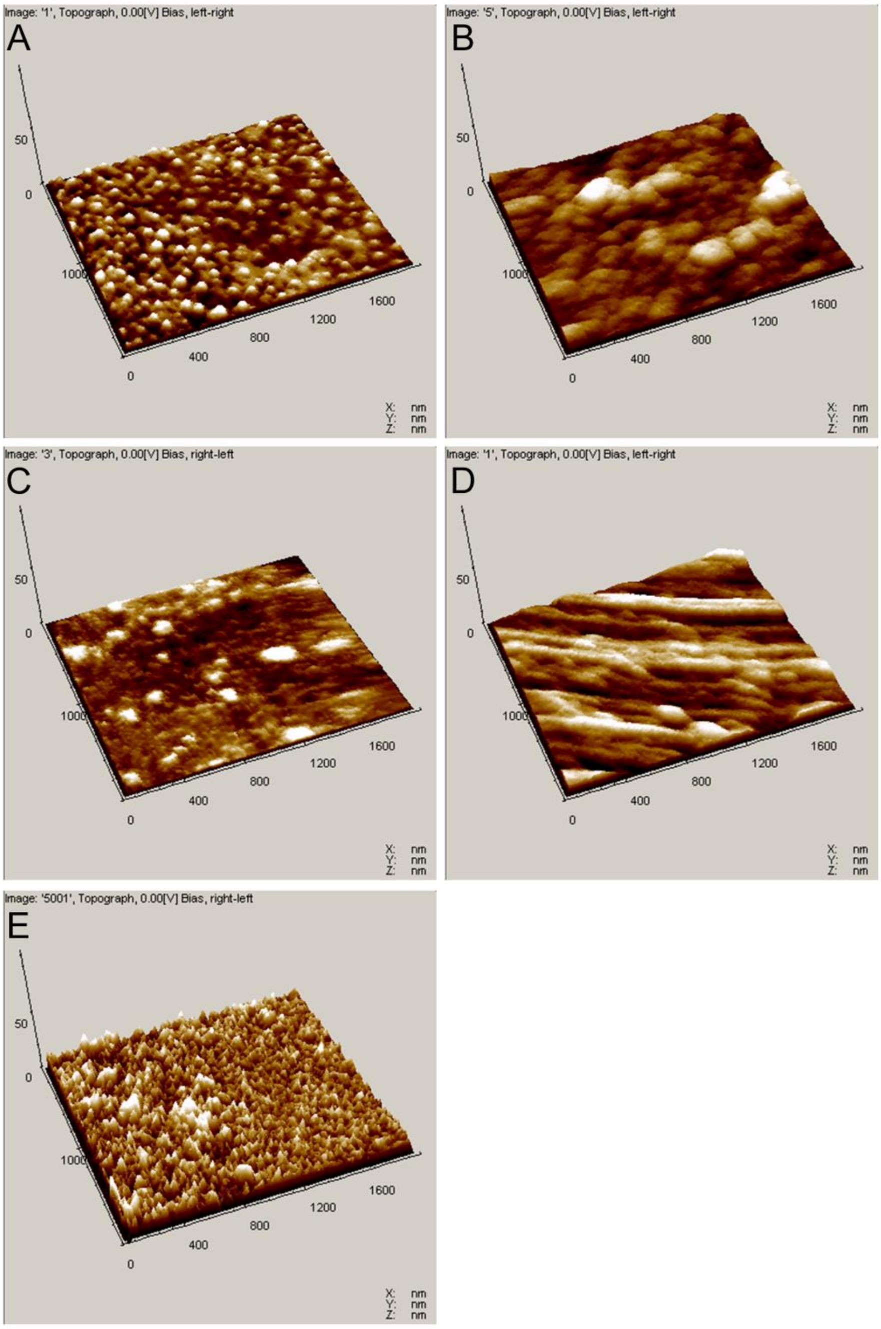

Supplementary FIGURE S3. Characterization of surface morphology by AFM. Representative height maps in three-dimensional view of the glass surface (**A**), PC surface (**B**), PVC surface (**C**), gold surface (**D**) and platinum surface (**E**).

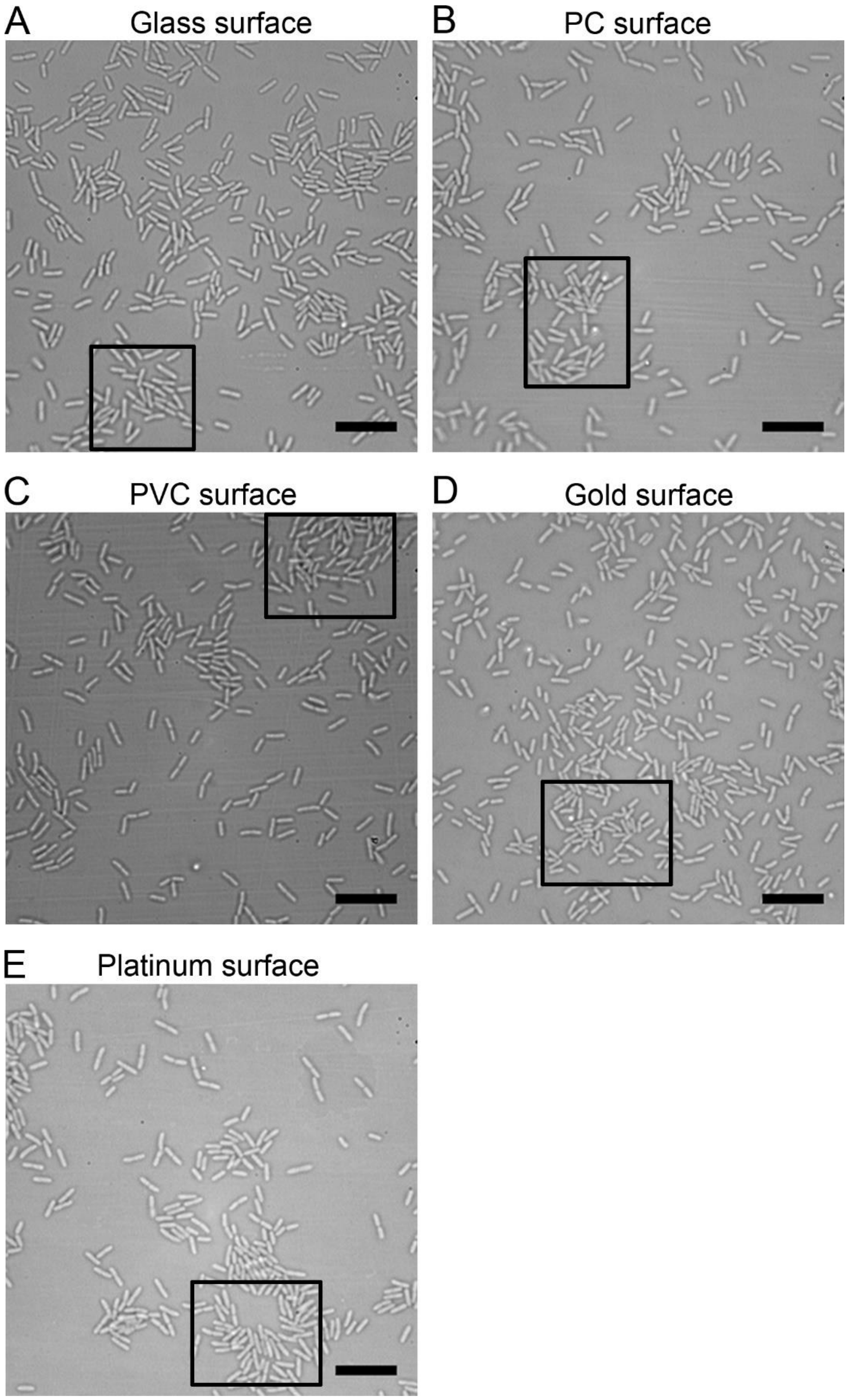

Supplementary FIGURE S4. More developed microcolonies of bacteria on the glass (A), PC (B), PVC (C), gold (D), and platinum (E) surfaces. Images were taken one hour later at the same locations as in Figure 3A. The regions outlined by black rectangles in the images confirm the growth of example microcolonies outlined by corresponding black rectangles in the bright-field images in Figure 3A. Scale bars are 10 μm.

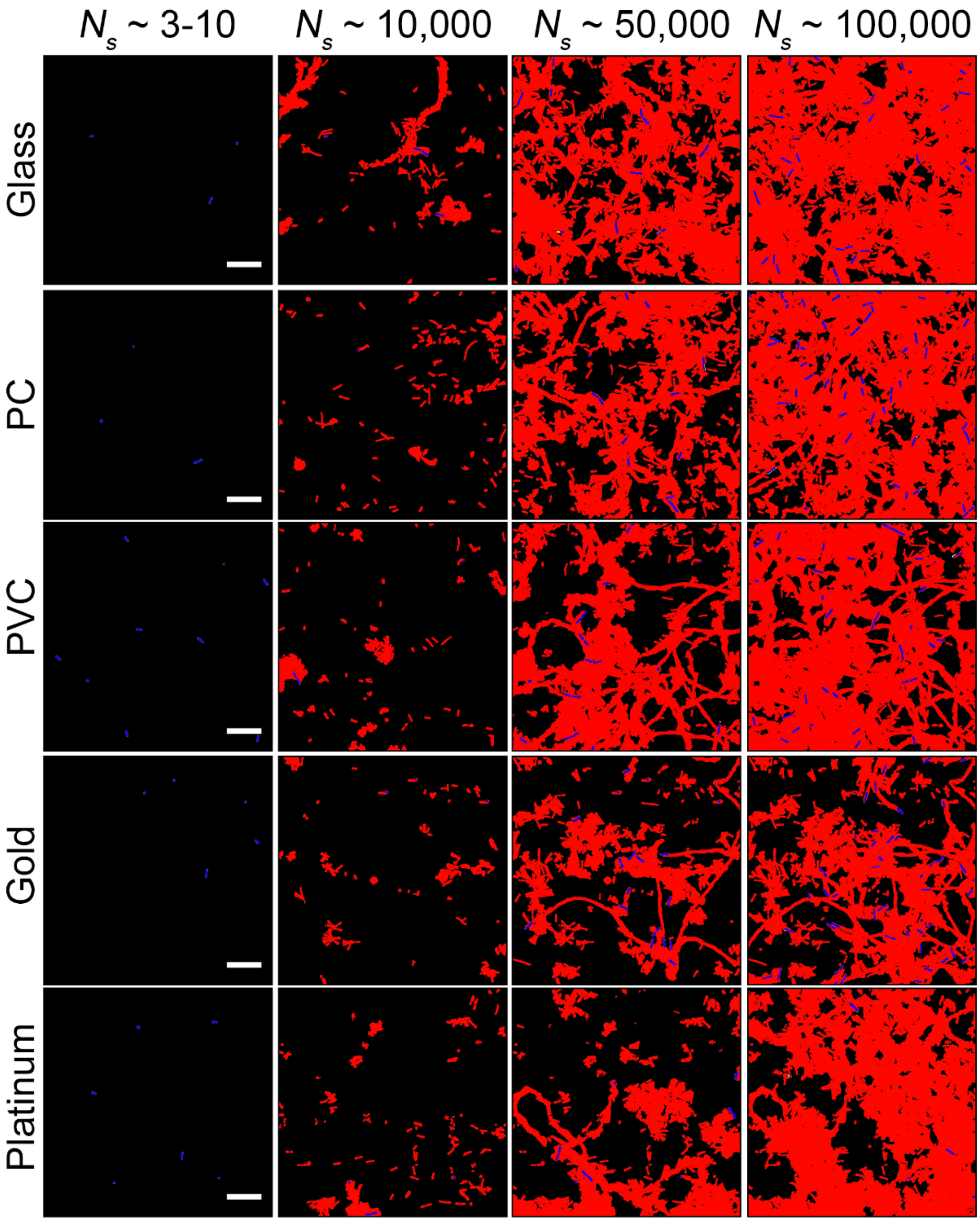

Supplementary FIGURE S5. Surface coverages for Psl- (△*pslBCD*) at different total number of bacterial visits N_s on the five tested surfaces. Red and black colors represent contaminated (*i.e.*, visited by bacteria) and fresh areas, respectively. Bacteria in the current frame are shown in blue. Scale bars are 10 µm.

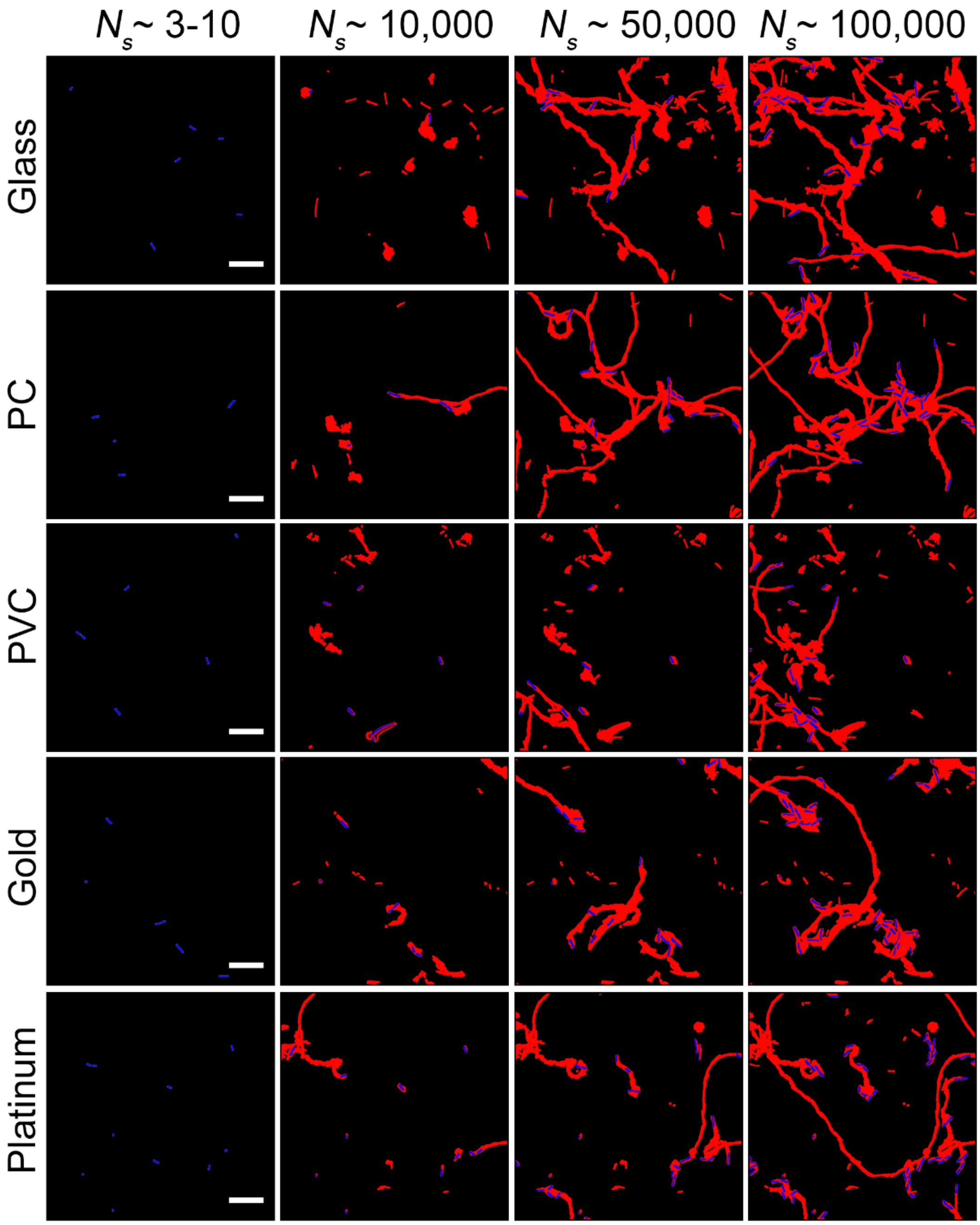

Supplementary FIGURE S6. Surface coverages for Psl++ (ΔP*psl*/P*BAD*-*psl* with 1% (w/v) arabinose addition in the medium) at different total number of bacterial visits N_s on the five tested surfaces. Red and black colors represent contaminated (*i.e.*, visited by bacteria) and fresh areas, respectively. Bacteria in the current frame are shown in blue. Scale bars are 10 μm.

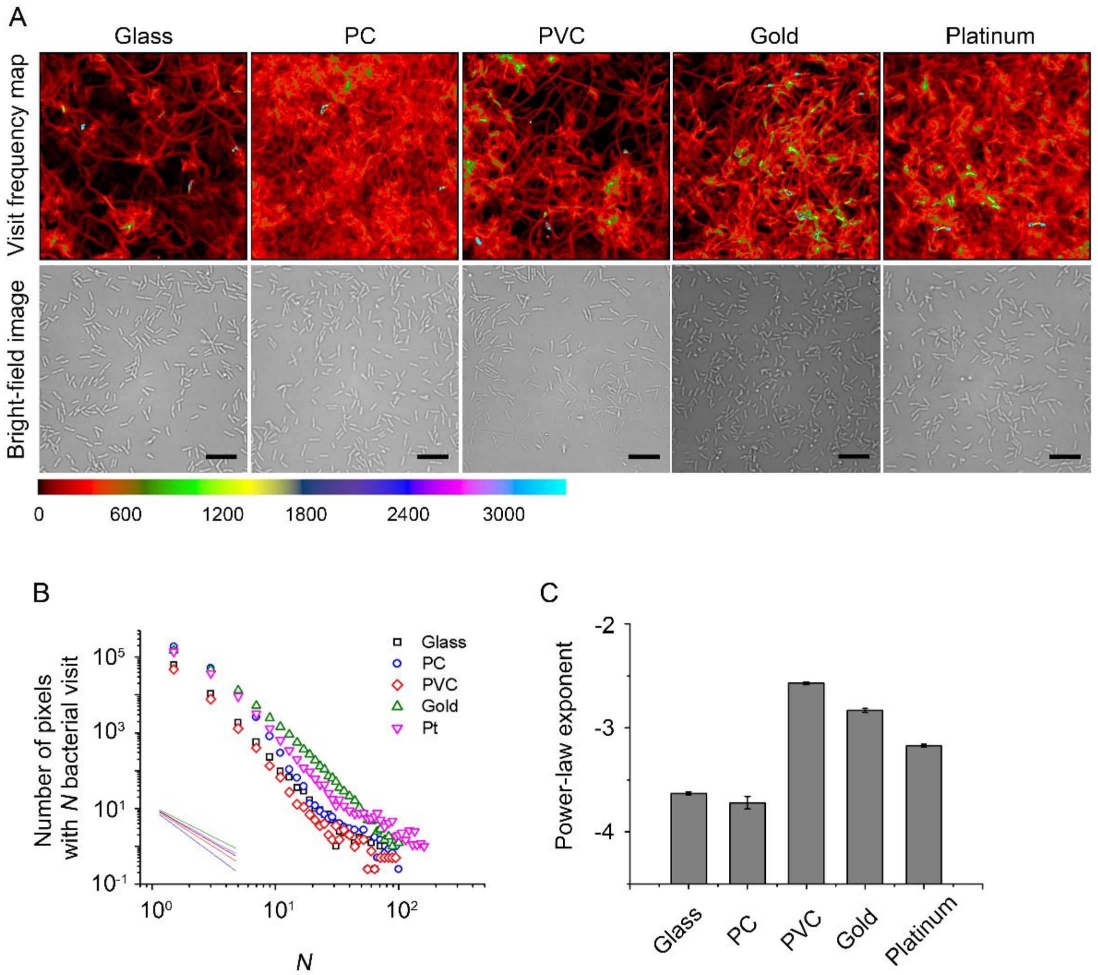

Supplementary FIGURE S7. Visit frequency distributions of Psl- (△*pslBCD*) on different surfaces. (**A**) Visit frequency maps (top row) and bright-field images (bottom row) at total bacterial visits of 1,140,300 for glass, 1,416,600 for PC, 1,356,100 for PVC, 1,089,000 for gold, and 554,500 for platinum, respectively, when microcolonies are just starting to form. (**B**) Visit frequency distributions. Solid lines show power-law decay. (**C**) Power-law exponents obtained from B. Error bars are standard deviations of three repeats. Scale bars are 10 μm.

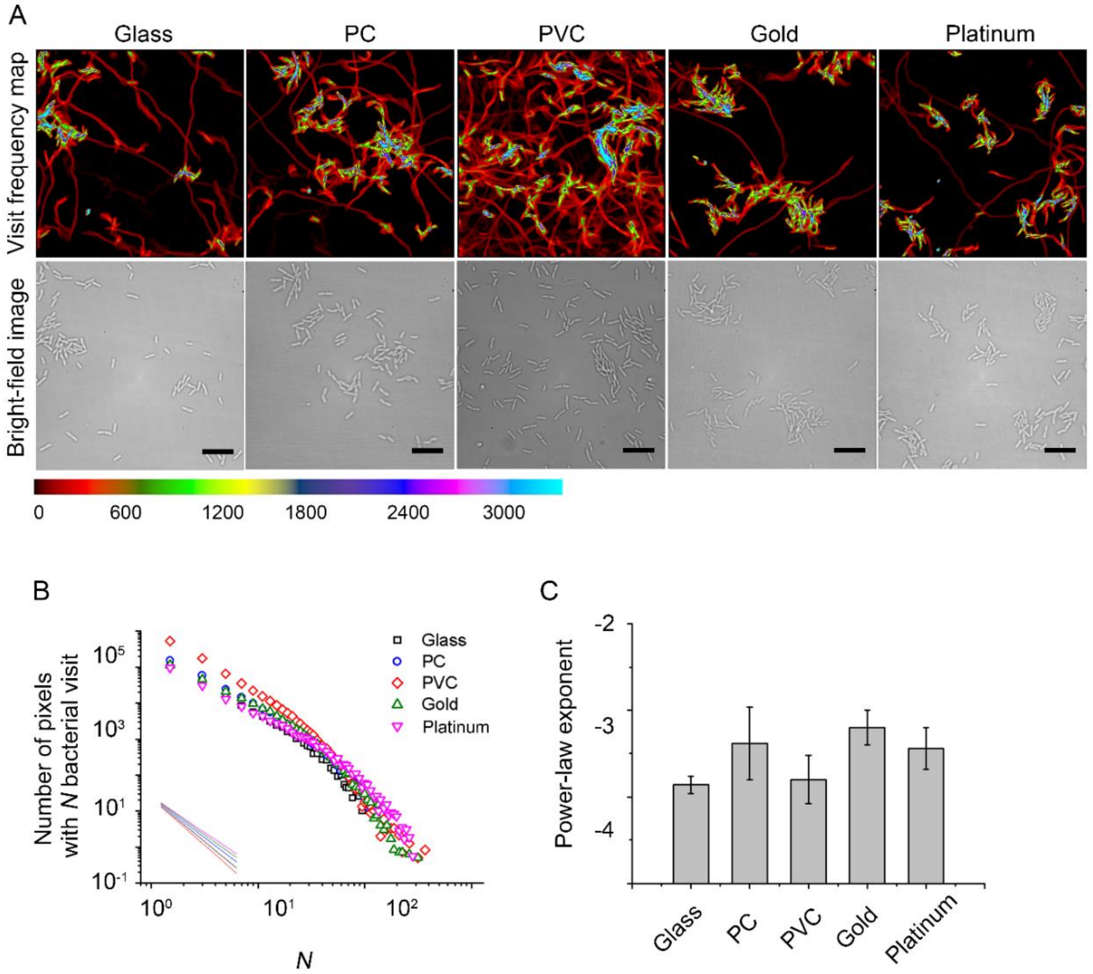

Supplementary FIGURE S8. Visit frequency distributions of Psl++ (△P_{psl}/P_{BAD}-*psl* with 1% (w/v) arabinose addition in the medium) on different surfaces. (**A**) Visit frequency maps (top row) and bright-field images (bottom row) at total bacterial visits of 149,600 for glass, 181,900 for PC, 199,400 for PVC, 221,700 for gold, and 213,200 for platinum, respectively, when microcolonies are just starting to form. (**B**) Visit frequency distributions. Solid lines show power-law decay. (**C**) Power-law exponents obtained from B. Error bars are standard deviations of three repeats. Scale bars are 10 μm.

Supplementary Table S1. Characterized surface properties of the five chosen surfaces

Surface	WCA [a] (°)	DCA [b] (°)	Surface energy (mJ/m^2)	Zeta potential (mV)	Ra[c] (nm)	Rq[d] (nm)
glass	32.1	52.9	63.9	-63.0	28.48	34.75
PC	86.4	22.8	47.7	-60.9	0.69	0.94
PVC	76.6	38.6	44.7	-53.9	0.72	1.00
gold nanofilm	34.7	48.6	64.2	-57.8	2.63	3.17
platinum nanofilm	36.1	41.8	65.2	-50.4	3.72	4.84

a) Water Contact Angle; b) Diiodomethane Contact Angle; c) average roughness; d) root-mean-square roughness
